# Retraction: Structural characterization and anticancer potency of centipede oligopeptides in human chondrosarcoma cancer: inducing apoptosis

**DOI:** 10.1039/d3ra90082g

**Published:** 2023-09-04

**Authors:** Yuebing Ren, Haibo Song, Yuanpeng Wu, Xiaochun Ma, Xuezhong Yu, Jia Liu, Jianhui Sun, Zhicheng Zhang

**Affiliations:** a Department of Cardiology, Dongying People's Hospital Dongying Shandong 257091 China zczhang_2019@163.com +86-546-83311902 +86-546-83311902

## Abstract

Retraction of ‘Structural characterization and anticancer potency of centipede oligopeptides in human chondrosarcoma cancer: inducing apoptosis’ by Yuebing Ren *et al.*, *RSC Adv.*, 2020, **10**, 29780–29788, https://doi.org/10.1039/D0RA04811A.

The Royal Society of Chemistry hereby wholly retracts this *RSC Advances* article due to significant portions of text overlap with a number of sources throughout the article, in particular ref. 13 and 14 of the original article and ref. 1–6 below, which were not cited.

The authors were informed about the retraction of this article but did not respond.

Signed: Laura Fisher, Executive Editor, *RSC Advances*

Date: 22nd August 2023

## Supplementary Material
